# Management of mixed urinary incontinence: IUGA committee opinion

**DOI:** 10.1007/s00192-023-05694-z

**Published:** 2024-01-22

**Authors:** Swati Jha, Peter C. Jeppson, Fulya Dokmeci, Gisele V. Marquini, Marair G. F. Sartori, Pamela Moalli, Shazia A. Malik

**Affiliations:** 1 Department of Urogynecology, Jessop Wing, Sheffield Teaching Hospitals NHS Foundation Trust & University of Sheffield, Sheffield, UK; 2 The Woman’s Center for Advanced Pelvic Surgery, The University of Arizona, Phoenix, AZ USA; 3 Department of Obstetrics & Gynecology, Ankara School of Medicine, Ankara University, Ankara, Türkiye; 4 Federal University of Uberlândia (UFU), Minas Gerais, Brazil and Federal University of São Paulo (UNIFESP), São Paulo, Brazil; 5 Urogynecology Division, Gynecology Department, Federal University of São Paulo, São Paulo, Brazil; 6 Division of Urogynecology and Reconstructive Pelvic Surgery, Department of Obstetrics, Gynecology and Reproductive Sciences, University of Pittsburgh, Pittsburgh, PA USA; 7 Female Pelvic Medicine & Reconstructive Surgery, Department of Ob/Gyn, University of Arizona COMPhoenix, Tucson, AZ USA

**Keywords:** Mixed urinary incontinence

## Abstract

**Introduction and hypothesis:**

Mixed urinary incontinence (MUI) is defined by the International Urogynecology Association (IUGA) and International Continence Society as the complaint of involuntary leakage of urine associated with urgency and also with exertion, effort, sneezing or coughing. It therefore implies the coexistence of both stress urinary incontinence (SUI) and urgency urinary incontinence (UUI). MUI is a heterogeneous diagnosis that requires an assessment of its individual components of SUI and UUI. Management requires an individualised approach to the symptom components. The aim of this review is to identify the assessment/investigations and management options for MUI.

**Methods:**

A working subcommittee from the IUGA Research & Development (R&D) Committee was created and volunteers invited from the IUGA membership. A literature review was performed to provide guidance focused on the recommended assessment and management of MUI. The document was then evaluated by the entire IUGA R&D Committee and IUGA Board of Directors and revisions made. The final document represents the IUGA R&D Committee Opinion.

**Results:**

The R&D Committee MUI opinion paper provides guidance on the assessment and management of women with MUI and summarises the evidence-based recommendations.

**Conclusions:**

Mixed urinary incontinence is a complex problem and successful management requires alleviation of both the stress and urge components. Care should be individualised based on patient preferences. Further research is needed to guide patients in setting goals and to determine which component of MUI to treat first. The evidence for many of the surgical/procedural treatment options for MUI are limited and needs to be explored in more detail.

## Introduction

Mixed urinary incontinence (MUI) is the involuntary loss of urine associated with urgency and physical exertion [1]. It is difficult to know whether MUI is a single entity with components of both urge and stress incontinence or whether it is actually two separate entities presenting simultaneously. Stress urinary incontinence (SUI) is a urethral problem attributed to pelvic floor weakness, functional loss of the urethral sphincter, and/or loss of the urethrovesical angle; whereas urgency incontinence is a bladder problem attributed to uninhibited bladder contractions.

Mixed urinary incontinence presents a complex clinical problem as successful treatment requires alleviation of both the stress and the urge components. The prevalence estimates of MUI vary widely based on symptom acquisition from 8.3% when based on objective urodynamics (UDS) findings to 93.3% when based on subjective patient-reported symptoms.

An important clinical dilemma is which component to treat first. Traditional thinking in the management of MUI involved treatment for whichever form of urinary incontinence (UI) predominates [2]. Unfortunately, women with MUI often have more severe symptoms and respond less well to treatment than women with isolated SUI or urgency urinary incontinence (UUI) [3]. Treatment of MUI is ultimately based on the composite assessment and shared decision making based on elicited patient expectations and treatment goals.

Many studies report on the independent treatment of either stress or UUI but surprisingly few studies report on the treatment of MUI. Throughout the remainder of this manuscript, we will discuss assessment options followed by conservative, pharmacological and then procedural treatments of MUI.

## Assessment of MUI

### History

The first step of evaluating MUI begins with a detailed history. A full assessment of urinary incontinence cannot be made in isolation and should include discussion of other pelvic floor issues including bladder, bowel, vaginal and sexual symptoms. For example, the impact of constipation and bowel problems on lower urinary tract symptoms is well established [4, 5]. It is important to discuss contributing risk factors including medical, obstetric, gynaecological and surgical history, as well as underlying neurological problems, incontinence surgery, drug therapy and prior radiation, all of which can have a significant impact when deciding on additional therapy.

Part of the discussion should focus on which component of MUI (stress or urgency) is the more bothersome symptom. This determination can be complemented by the use of validated patient-reported outcome measures (PROMs) as these allow a more accurate subjective assessment of women with urinary incontinence. There is a wide range of PROMs that assess many domains of pelvic floor function [6]. Some of these questionnaires assign a score to the different components of lower urinary tract dysfunction, which allows the clinician to assess the severity and degree of bother caused by the individual symptoms including stress or urgency incontinence.

### Bladder diary

The frequency volume chart/bladder diary is recommended by the International Continence Society and the European Urology Association [7, 8] as a part of routine workup. The electronic bladder diary, first described in 2003 [9] allows more objective symptom assessment. There is some evidence to suggest that electronic diaries might be more preferable to patients irrespective of gender, age or education [10].

The optimal duration of a bladder diary is usually accepted as 3 days [11]. The 3-day diary has been validated to provide data comparable with a 7-day diary and is less burdensome to the patient. Patients are asked to record the number, time and volume of each micturition, urgency and incontinence episodes including what activities were temporally associated with the leakage.

### Physical examination

A detailed pelvic and abdominal examination should be performed noting examination findings such as pelvic or abdominal masses, vaginal atrophy, pelvic muscle strength and vaginal prolapse as these conditions are associated with MUI. Vaginal atrophy is easily treatable if detected and has been associated with urinary incontinence [12].

Although pelvic floor muscles can be assessed by a variety of scales, we suggest using the Oxford grading system, which consists of evaluating pelvic muscle strength with digital palpation during a maximal voluntary contraction on a scale of 0 to 5 [13]:

0/5 No contraction
1/5 Visible/palpable muscle contraction but no movement
2/5 Movement with gravity eliminated
3/5 Movement against gravity only
4/5 Movement against gravity with some resistance
5/5 Movement against gravity with full resistance

Physical examination can also assess urethral mobility as hyper- or hypo-mobility can impact treatment options. Mobility of the bladder neck is assessed based on the difference between the urethral position at rest and with Valsalva and can be done using perineal/translabial ultrasound (objective assessment) or simple observation (subjective assessment). The Q tip test is now outdated. Studies have shown that subjective and objective assessments of urethral hypermobility are comparable [14].

## Investigations

### Urodynamics

Evidence supporting the role of UDS prior to the invasive treatment of MUI is not robust. There are studies that have failed to find a corelation between the need for re-intervention following surgery for MUI and preoperative UDS testing [15]. Conversely, another study concluded that women who received treatment concordant with their UDS findings were more likely to report an improvement in bladder symptoms [16]. Many providers obtain UDS evaluation prior to performing invasive therapy for urinary incontinence. Ideally, UDS would demonstrate evidence of both SUI and detrusor overactivity (DOA) to make a diagnosis of MUI. During UDS, it is important to identify cough-induced DOA as this can be confused with MUI [17].

### Ultrasound scan (transperineal/translabial)

Ultrasound evaluation is not standard practice in every setting, but is the source of ongoing clinical evaluation. Transperineal/translabial ultrasound [18] in women with stress-predominant MUI provides information regarding dynamic posterior urethral angle, dynamic angle of urethral inclination, descent of the bladder neck and dynamic pubourethral distance. In a study of women with MUI, those with urge-predominant MUI had a greater detrusor thickness; and those with equal stress and urge had a greater descent of the bladder neck (*p* < 0.05). In this study UDS and ultrasound parameters differed significantly between the stress-predominant and urge-predominant groups (*p* < 0.05) suggesting that these might be distinct entities. For example, the dynamic angle of urethral inclination, descent of the bladder neck and dynamic pubourethral distance correlated inversely with detrusor pressure at maximal flow and functional urethral length, whereas detrusor wall thickness correlated positively with detrusor pressure at maximal flow and functional urethral length. Comparing transperineal ultrasound with UDS, ultrasound was tolerated better [18].

## Treatments

### Conservative treatment of MUI

For this manuscript, we define conservative treatment as non-pharmacological and non-surgical approaches, which are generally considered first-line treatment options. Counselling/cognitive therapy, lifestyle modifications, scheduled voiding, pelvic floor muscle training (PFMT), mobile health apps, electroacupuncture (EA) and urgency suppression techniques are all examples of preliminary therapies designed to improve bladder control while promoting a healthy lifestyle. Conservative treatment has varied levels of evidence, but is recommended as a first-line therapy owing to its high safety profile with limited risks [19–21].

### Counselling/cognitive therapy

Cognitive behavioural therapy (CBT) is based on the concept that thoughts, feelings, physical sensations and actions are interconnected and works by changing the way in which patients think and behave. In a systematic review assessing the impact of CBT, there was a high level of evidence for the effectiveness of CBT on symptom severity and a moderate level of evidence for the effectiveness of CBT on quality of life, psychological symptoms and patient satisfaction. However, there is no robust evidence favouring an impact of CBT on the clinical signs of incontinence [22].

### Lifestyle modifications

1. Fluid consumption/restriction: usually recommended together with scheduled voiding
2. Dietary modification: high-fiber foods recommended to avoid constipation
3. Elimination diet: for urinary urgency incontinence to reduce bladder irritants
4. Physical activity: physical exercises suggested to improve muscle volume and blood flow into the pelvis

A 2015 Cochrane review that included 11 studies with 5,974 predominantly female participants provided low-level evidence for the effect of commonly suggested lifestyle alterations on all types of urinary incontinence such as weight loss, fluid intake and caffeine reduction. The authors reported that no eligible trials were identified that investigated the effects of smoking cessation, reduction of alcohol intake, avoidance of straining and constipation, and level of physical activity on urinary incontinence [23].

However, based on systematic reviews and meta-analysis, non-surgical weight loss intervention was reported as a critical part of UI treatment as it improves symptoms of urinary incontinence frequency and urgency in overweight women [24]. Hence, lifestyle interventions as initial therapy for MUI can be recommended on an individualised basis.

### Scheduled voiding regimes

1. Bladder training
2. Timed voiding
3. Habit voiding
4. Prompted voiding

Bladder training is often used in conjunction with other lifestyle modifications, to correct habitual frequent urination by gradually increasing intervals between voids. Urgency suppression techniques facilitate the ability to cope with delayed voiding. Techniques include distraction, relaxation, deep breathing, perineal pressure, toe curling, plantar flexion of the foot and pelvic floor muscle contraction [19]. Bladder training lasting for at least 6 weeks with other lifestyle interventions, such as caffeine reduction, weight loss and modified fluid intake, is recommended as initial therapy for all women with MUI, with strong supporting evidence [20, 21]. Moderate- to high-level evidence supports that bladder training cures or improves UUI symptoms in more women compared with no intervention and combining it with PFMT results in more improvement or cure than bladder training alone in women with all types of urinary incontinence including MUI [25].

### Pelvic floor muscle training and adjunctive therapies

Pelvic floor muscle training is the most widely recommended first-line treatment for all types of UI in women. Adjunctive or secondary therapies such as biofeedback-assisted PFMT and neuromuscular electrical stimulation have been shown to be superior to PFMT alone. Recently, a Cochrane systematic review focusing on conservative interventions for women with all types of UI, analysed 29 systematic reviews that included 112 trials with 8,975 women [25]. This study provided moderate- to high-level evidence derived from 13 of the systematic reviews that PFMT, electrical stimulation, weight loss, and vaginal cones improved chance of cure or improvement of all types of UI compared with control. High-level evidence derived from this study supported the widespread recommendations that conservative treatment plans should prioritise PFMT for women with all types of UI, including MUI. Moreover, intense and frequent PFMT with supervision and combined with lifestyle modifications were more effective in terms of reduced incontinence episodes and improved quality-of-life measures. Electrical stimulation was reported to be more effective in women with UUI than in inactive intervention and likewise cones were found to be more beneficial than no intervention for women with SUI regarding symptomatic relief with a moderate level of evidence [25].

### Mobile health applications (mHealth app)

Use of mHealth apps among women who need PFMT provides not only time-saving and cost benefits but was also found to be helpful for long-term treatment adherence that is essential for treatment success. In a systematic review of 6 RCTs comparing the use of an mHealth app-based approach vs PFMT alone in women with SUI or stress-predominant MUI, women treated using the mHealth app had better improvement in terms of symptom severity and exercise adherence [26]. However, it is noteworthy to emphasise that all available mobile health technologies for the management of UI may not provide high scores in terms of credibility, user interface, experience and engagement [27].

### Urgency suppression techniques

The Knack tutorial is a self-administered, vignette-based instructional programme on pre-empting bladder challenges in daily life (urgency, stress-leakage, or urge leakage) through anticipatory, well-timed pelvic floor muscle contraction at the moment of challenge. In a randomised controlled trial [28] of 108 women with stress or MUI, 64 women were randomised to a Knack tutorial group with a 15-min slide show, with 10 vignettes portraying the use of the Knack in daily life, and 59 women were randomised to a diet/exercise tutorial group. Self-perceived improvement was reported in 71% of women in the Knack tutorial group and 25% in the diet/exercise group, 1 month after viewing the tutorial (*p* < 0.001). An electronic version of this tutorial is now available to help women with MUI to overcome the daily life challenges associated with urgency and UUI and may become more effective if incorporated into eHealth apps (http://www.myconfidentbladder.com).

### Pharmacological treatment

A systematic review of the non-surgical treatment options for women with urinary incontinence was published in 2019 [29]. That review identified 84 studies, of which only 4 reported specifically on the treatment of MUI. Because of the lack of direct comparisons for the treatment of MUI, a network meta-analysis was performed to directly and indirectly compare interventions. As the search for that systematic review was completed in 2018, we updated a search specific to pharmacological therapy to capture any additional studies published after that. Twelve new studies were identified [30–41]. We include the outcomes reported in that systematic review coupled with a narrative report of the information from the 12 newer studies; we did not update that systematic review or perform an updated network meta-analysis with all studies.

Pharmacological options for the treatment of MUI are as follows:

1. Antimuscarinics/anticholinergics
2. Alpha-agonists
3. Beta-agonists
4. Oestrogens
5. Tricyclic antidepressants (combined alpha-agonists and anticholinergic)

#### Antimuscarinics/anticholinergics

Antimuscarinic and anticholinergic medications block bladder receptors, resulting in decreased bladder overactivity and therefore may improve symptoms of UUI. Many medications in this category have been developed, such as darifenacin, fesoterodine, flavoxate, oxybutynin, phenylpropanolamine, pilocarpine, propantheline, propiverine, solifenacin, tolterodine and trospium [29]. These medications have been shown to be effective for both cure (OR 1.95) and improvement (OR 2.95) of UUI symptoms versus no treatment (OR 1.95) [29].

Three studies evaluated medications for the treatment of MUI [34, 35, 41]. A study from Hong Kong in which women were treated with antimuscarinic medication demonstrated that 58 out of 87 women (29.3%) showed subjective improvement in both SUI and UUI symptoms [41]. Women with UUI-predominant MUI symptoms also demonstrated improvement (77.6% vs 22.4%, *p* = 0.001). A study from Japan demonstrated that propiverine hydrochloride resulted in decreased rates of both UUI and SUI of 63.9% and 44.3% respectively [34]. Propiverine appears to provide effective therapeutic benefit for patients with MUI, although the efficacy appeared greater for the UUI than for the SUI component.

A prospective, single-blind randomised controlled study on 60 women with MUI that received either mace powder (plant extract with anticholinergic properties) versus placebo, reported cure rates of 90% for the mace group versus 16.66% for the control group (*p* < 0.001) with no side effects [35].

A multicentre randomised controlled trial was conducted in China comparing solifenacin with EA for women with MUI [32]. The reduction in incontinence episodes at 12 weeks was comparable between groups, 38% in the EA group, and 37% in the solifenacin group (*p* < 0.001 for non-inferiority).

#### Alpha-agonists

Serotonin and norepinephrine reuptake inhibitors (SNRI) are thought to have an alpha agonist effect on the bladder and urethra, and may thereby improve symptoms of MUI. The reflexes of continence and micturition are modulated by serotonin, which increases urethral closure and simultaneously reduces the micturition reflex [42].

Alpha agonists such as duloxetine, and midodrine were found to be more effective than no treatment for improvement of MUI (OR 2.16) [29]. Compared with hormone therapy, alpha agonists were found to be much more effective for improvement of MUI (OR 4.09).

Duloxetine, a SNRI, is an approved treatment for SUI. A placebo-controlled double-blind study of women with MUI showed a significant reduction in UUI and SUI episodes comparing duloxetine and placebo. The best results were seen in patients with stress-predominant symptoms [42, 43]. Women with MUI treated with 40 mg of duloxetine daily were found to have a 62% reduction of incontinence episodes [42]. However, duloxetine has been associated with adverse events such as nausea, emotional alterations, violence, depression, suicidal ideation and attempted suicide [44]. So, the benefits of these medications need to be balanced against the risks.

Litoxetine is a selective and specific SNRI and multifunctional serotonin agonist-antagonist that has demonstrated increased bladder capacity and urethral sphincter pressure, in non-clinical studies [31].

A recent paper described two randomised controlled studies that were conducted to evaluate the effect of litoxetine compared with placebo in patients with MUI. The first study did not meet the primary efficacy endpoint. The second study showed no difference in frequency of adverse effects, and litoxetine reduced the number of incontinence episodes. Based on these results, litoxetine may be a safer and more tolerable treatment for MUI than duloxetine [30].

#### Beta-agonists

β3-adrenoceptor agonist medications work by interfering with bladder neuroreceptors, resulting in decreased bladder overactivity. These have been studied in women but were not included in the 2018 systematic review, as studies were mixed gender and did not have a high enough percentage of women to be included in that review [29].

Our updated search identified one post hoc analysis of two RCTs conducted in Japan, including 261 women with MUI treated with 50 mg of mirabegron versus placebo. This analysis demonstrated improvement in incontinence episodes (*p* < 0.001), voided volume (*p* = 0.005), and nocturia episodes (*p* = 0.03) [38]. A systematic review on the efficacy of vibegron (β3-adrenoceptor agonist) included 3 RCTs and demonstrated improved urinary frequency, urgency and UUI episodes (*p* < 0.0001) [37].

#### Oestrogens

Compared with no treatment, hormone therapy, including topical oestrogen and raloxifene, was found to be effective treatment for MUI (OR 2.89) [29].

There is no consensus on the action of vaginal or systemic oestrogens in the treatment of urge or stress incontinence [45]. Moreover, there is a lack of evidence regarding dosing, time of use, or oestrogen types used for the treatment of UI [46].

A Cochrane review suggests topical vaginal oestrogen may improve urinary incontinence, while systemic oestrogen worsened incontinence symptoms (risk ratio (RR) 1.32, 95% CI 1.17 to 1.48) [47]. The same result was observed in another study, with 1-year follow-up, in which there was worsening incontinence (RR 1.11, 95% CI 1.04 to 1.18) [48]. Therefore, it seems that systemic oestrogen (oral or transdermal) may increase the risk of UI [49].

In the comparison of hormonal therapy and surgery, one study assessed the effectiveness of ospemifene in the improvement of the urgency component in women with MUI who underwent surgery with a midurethral sling (MUS). They concluded that ospemifene improved urgency symptoms and quality of life after surgery in women with MUI [36].

In conclusion, topical hormonal therapy seems to improve symptoms of MUI and may be a treatment option for selected patients.

#### Tricyclic antidepressants

Imipramine is a tricyclic antidepressant drug with dual drug class effects with both antimuscarinic and alpha agonist properties [50].

To our knowledge, there are no randomised controlled trials studying the effects of imipramine in women with MUI. Some open-label studies have shown some beneficial effects in SUI [51]. However comparative studies suggest variable success rates for MUI, with minimal improvement in SUI, and some studies showing no improvement in UUI whereas others show improvement rates of UUI ranging from 44 to 90% [52, 53].

Side effects of imipramine can limit the use of this drug, especially the possibility of arrhythmias. It should not be prescribed to patients with psychiatric disorders, patients who use monoaminoxidase inhibitors, or those at an increased risk of arrhythmias [54].

### Surgical management of MUI

Surgical options for the treatment of MUI include interventions aimed at either SUI or UUI. Options for SUI include MUS (retropubic, transobturator, or single incision), Burch colposuspension, pubovaginal slings and urethral bulking agents. Procedures for UUI include intradetrusor onabotulinum toxin A, acupuncture, EA, or sacral neuromodulation.

There are no international published guidelines that dictate whether it is better to start with surgical management to address the SUI or UUI component of MUI although the usual recommendation is to start with less invasive therapies when managing MUI, as this has the potential to avoid surgery altogether [55]. Within treatment regimens, surgical success is largely dependent on the degree of pre-existing UUI symptoms, as overall improvement in MUI is dependent on improvement in UUI symptoms [2, 56–59]. Studies have shown that initial improvements in UUI may decline with time [60, 61].

#### Midurethral slings

Since Ulmsten et al. introduced the idea of a retropubic sling in 1996, both retropubic and transobturator slings have proven to be gold-standard MUS treatments for SUI [62] although studies suggest a slight benefit of the retropubic sling [63].

Long-term data have supported the durability of MUS for MUI, revealing a significantly improved quality of life in 85.3% of patients [64]. Risk factors for persistent UUI postoperatively include age greater than 60 and post-menopausal status [63]. Single-incision slings are effective for the treatment of SUI but have not shown to be as effective for MUI. In one particular study following patients 2 years post-operatively, the subjective cure rate was significantly lower in the single-incision sling arm than in the MUS arm (55.3% vs 84.0% respectively; RR = 0.66; 95% CI, 0.54–0.80]; *p* < 0.001). The proportion of retreated patients for SUI/MUI was significantly higher in the single-incision arm than in the MUS arm (34.9% vs 11.3% for the MUS arm; *p* < 0.001) [65].

Persistent urinary urgency after MUS treatment is prevalent and ranges from 30 to 70% [66, 67]. Overall success rates following MUI in patients with MUI are lower than in those with pure SUI [68]. MUS in combination with pelvic floor muscle therapy can improve MUI symptoms [69].

In a secondary analysis of data from three multi-centre surgical trials of women with stress-predominant MUI assigned to Burch colposuspension, autologous fascial pubovaginal sling, retropubic MUS, or transobturator MUS, significant improvements in irritative/storage symptoms were reported by all surgical groups 1 year after surgery [61].

#### Burch colposuspension

Mixed urinary incontinence treated by Burch colposuspension seems to result in improved SUI and UUI symptoms based on secondary analysis from multicentre studies [61, 70]. Open and laparoscopic colposuspension have been shown to be equally effective surgical treatments for SUI in the short term [71], with cure rates reported as 85–90% at 1 year decreasing to 70% at 5 years. Laparoscopic colposuspension seems to have similar subjective outcomes to retropubic MUS in the management of MUI based on the limited evidence available [56].

#### Autologous fascial pubovaginal sling

The autologous fascial pubovaginal sling (AFPS) is an effective and durable treatment for SUI, both as a primary procedure and a secondary surgery for those who failed other anti-incontinence surgeries. The AFPS appears to be as effective as MUS for the treatment of SUI in the short term [72]. In a retrospective observational study, women with MUI demonstrated comparable improvement to women with isolated SUI suggesting AFPS can be a treatment for MUI [73]. This study demonstrated that persistent episodes of urgency and urgency incontinence noted on the preoperative voiding diary correlated directly with surgical failure, while voiding frequently was associated with cure [73].

#### Urethral bulking agents

Periurethral bulking agents can be used to improve urethral coaptation by adding a mass effect to the urethra, thereby decreasing UI. This is a relatively quick, minimally invasive procedure that can be done in an office setting or in the operating room. Although the cure rate of urethral injection therapy is lower than for synthetic MUS, it is an attractive option, particularly for women that are not candidates for more invasive surgical intervention [74, 75]. Previously used bulking agents included autologous fat, carbonated beads, collagen, dextranomer hyaluronate, polydimethylsiloxane and porcine collagen. Periurethal bulking agents were included in a systematic review and network meta-analysis of non-surgical treatments for UI. Indirect evidence found no improvement in periurethral bulking agents over no treatment (low strength of evidence) and they were less likely to result in cure than behavioural therapy (OR 0.23, 95% CI 0.06–0.98). In addition, serious adverse events were noted with periurethral bulking agents, with an erosion rate and a need for surgical removal of the injectant in 4.7% based on data from 362 women in 3 studies [76].

Polyacrylamide hydrogel (PAHG) has been available in Europe for the past decade and was approved for use by the FDA in the USA in 2020. A study from Denmark to assess the cure rate of PAHG for MUI when it was first introduced revealed a 2-year subjective cure rate of only 29.8–36.1% [77]. A subsequent 7-year follow-up study has demonstrated cure rates of 67.1% [78]. Cure rates were worse for those with strong urinary urgency symptoms [77, 79].

### Other procedures

#### Onabotulinum toxin A

Intradetrusor onabotulinum toxin A (Botox; Allergan/Dysport, Ipsen) has been used primarily to target urinary urgency, frequency and urgency incontinence symptoms. One unit of Botox is equivalent to approximately 3–5 units of Dysport; therefore, a typical dosing of 100–200 units of Botox corresponds to 300–500 units of Dysport [80].

Studies involving onabotulinum toxin A treatment for MUI only involve combination therapies. A recent randomised controlled trial [81]: evaluated whether retropubic MUS combined with onabotulinum toxin A was more effective than sling alone in improving MUI symptoms. The outcomes showed that women with MUI undergoing sling application reported significant improvement in overall incontinence symptoms, regardless of the addition of onabotulinum toxin A injections (83% vs 84%). However, those receiving concurrent onabotulinum toxin A injections reported less urgency severity and greater improvement in urgency symptoms at 3 months. Other short-term studies have shown similar results by combining MUS with onabotulinum toxin A (100 IU) [80, 82].

One study indicated that the combination of onabotulinum toxin A and PAHG therapy may be an effective treatment for MUI in the elderly and frail population. At 12 months, objective and subjective cure rates were 50% and 40% respectively. In addition, patients benefited from a short surgical procedure without the need for general anaesthesia or discontinuation of anticoagulation [40].

#### Vaginal laser therapy

Erbium-doped yttrium aluminum garnet (YAG) laser therapy has been studied for the potential improvement of UI. Recent literature has shown that YAG laser therapy does not improve MUI symptoms [83, 84].

#### Electroacupuncture

In a secondary analysis of a non-inferiority trial comparing the safety and effectiveness of 12 weeks of EA with 36 weeks of PFMT plus solifenacin in 79 women with balanced MUI, the authors reported that EA was non-inferior to combined therapy as regards both symptom relief and increased quality of life. EA, however, demonstrated better safety [85]. An ongoing three-armed RCT investigating the effect of EA versus Sham (superficial needle insertion and no manipulation) versus no intervention for stress-predominant MUI may provide more data regarding the effectiveness of EA in the management of MUI [86].

The additive effect of PFMT when combined with other active therapies in the treatment of women with SUI, UUI and MUI was investigated in a Cochrane review that included 13 trials comparing 585 women undergoing PFMT plus another intervention and 579 women on another active treatment alone. Other active treatments include physical therapies (e.g. vaginal cones); lifestyle modifications; scheduled voiding (bladder training); electrical or magnetic stimulation; mechanical devices (e.g. continence pessaries); drug therapies (e.g. anticholinergics and duloxetine); and surgical interventions including sling procedures and colposuspension. Owing to a lack of evidence of a benefit, the additional effect of PFMT in treating SUI, UUI and MUI [69, 87] remains inconclusive. Further studies are needed to comment reliably on the beneficial effect of combined therapies for the treatment of MUI.

#### Sacral neuromodulation

A recent systematic review by Balk et al. found evidence that neuromodulation, which is typically used for the treatment of UUI, was more effective (OR 3.34, 95% CI 2.12–5.26) than no treatment for women with UI (high strength of evidence), which suggests that neuromodulation, which is effective for UUI, could be as effective for MUI [29]. The effect on the SUI component has been poorly studied.

Specifically, neuromodulation and onabotulinum toxin A were more effective than no treatment for MUI and onabotulinum toxin A may be more effective than neuromodulation (low level of evidence [OR 1.69; 95% CI 0.80–3.62]).

#### Posterior tibial nerve stimulation

Studies evaluating the benefits of posterior tibial nerve stimulation (PTNS) are in combination with MUS. According to a retrospective analysis of women affected by MUI with a predominant SUI component, MUS combined with PTNS versus MUS alone was more effective in treating MUI symptoms at a 3-month follow-up. The evidence is very limited and represents only a short-term follow-up [88].

#### Acupuncture

Previous studies showed some effects of acupuncture for MUI. In a recent systematic review utilising ten major databases, three randomised studies with 591 women were included. Acupuncture shows some benefit for women with MUI. The effect of EA on the reduction of numbers of incontinence, urgency and nocturia episodes was significant relative to the effect of tolterodine or solifenacin alone. More evidence is required to draw a solid conclusion of effectiveness and safety of acupuncture for women with MUI [89].

## Future research

There remain multiple unanswered questions in relation to the diagnosis and treatment of MUI. Research into the clinical utility of establishing the individual components and which to treat first, as well as methodology for patient goal setting and attainment is urgently needed. The evidence for many of the surgical/procedural treatment options for MUI is relatively poor and needs to be explored in more detail. The role of epigenetics and urinary microbiome in causation is poorly understood and needs further investigation. Further research into the natural progression, transition, prevention and risk factors for urgency incontinence may allow targeted treatment strategies to be implemented.

## Conclusion

- Mixed urinary incontinence is a complex problem and successful management requires alleviation of both the stress and urge components. Care needs to be individualised to patient preferences.
- The diagnosis is based on symptom assessment through a detailed history assisted by bladder diaries and examination. Investigations such as UDS are usually reserved for patients with refractory symptoms or those who choose invasive treatment. There is some evidence to support the use of ultrasound for non-invasive assessment.
- Conservative treatments are the recommended preliminary treatment owing to their high safety profile. Counselling/cognitive therapy, lifestyle modifications, scheduled voiding, PFMT, mobile health apps, EA and urgency suppression techniques are all recommended as first-line treatments to improve bladder control while promoting a healthy lifestyle.
- Pharmacological treatments are the usual treatment following failed conservative therapy and include antimuscarinics/anticholinergics, beta-agonists, topical oestrogens (in postmenopausal women), alpha-agonists, tricyclic antidepressants (combined alpha-agonist and anticholinergic), and botulinum toxin A.
- Surgical/procedural options for the treatment of MUI include interventions aimed at either SUI or UUI. Options for SUI include MUS (retropubic, transobturator or single incision), Burch colposuspension, pubovaginal slings and urethral bulking agents. Procedures for UUI include intradetrusor onabotulinum toxin A, vaginal laser, acupuncture, EA, PTNS, or sacral neuromodulation.
- As there is a risk of iatrogenic UUI post-SUI surgical procedures, usual practice would be to treat the UUI and achieve better control of the urgency symptoms prior to surgical treatment for the SUI component.
- Before commencing treatment, it is important to explore patients’ goals and goal attainment, as this may provide useful information to guide treatment-related decisions.
- Further research into several aspects of MUI is required, including the clinical utility of establishing the individual components and which to treat first, as well as methodology for patient goal setting and attainment. The evidence for many of the surgical/procedural treatment options for MUI is relatively poor and needs to be explored in more detail.

## References

[1] Haylen BT, de Ridder D, Freeman RM (2010). An International Urogynecological Association (IUGA)/International Continence Society (ICS) joint report on the terminology for female pelvic floor dysfunction. Neurourol Urodyn.

[2] Nightingale G (2020). Management of urinary incontinence. Post Reprod Health.

[3] Myers DL (2014). Female mixed urinary incontinence: a clinical review. JAMA.

[4] Alhababi N, Magnus MC, Drake MJ, Fraser A, Joinson C (2021). The association between constipation and lower urinary tract symptoms in parous middle-aged women: a prospective cohort study. J Womens Health (Larchmt).

[5] Carter D, Beer-Gabel M (2012). Lower urinary tract symptoms in chronically constipated women. Int Urogynecol J.

[6] Gray TG, Vickers H, Krishnaswamy P, Jha S (2021). A systematic review of English language patient-reported outcome measures for use in urogynaecology and female pelvic medicine. Int Urogynecol J.

[7] Abrams P, Andersson KE, Apostolidis A (2018). 6th International Consultation on Incontinence. Recommendations of the International Scientific Committee: evaluation and treatment of urinary incontinence, pelvic organ prolapse and faecal incontinence. Neurourol Urodyn.

[8] Nambiar AK, Arlandis S, Bo K (2022). European Association of Urology Guidelines on the diagnosis and management of female non-neurogenic lower urinary tract symptoms. I. Diagnostics, overactive bladder, stress urinary incontinence, and mixed urinary incontinence. Eur Urol.

[9] Quinn P, Goka J, Richardson H (2003). Assessment of an electronic daily diary in patients with overactive bladder. BJU Int.

[10] Palmer C, Farhan B, Nguyen N (2018). Are electronic and paper questionnaires equivalent to assess patients with overactive bladder?. J Urol.

[11] Konstantinidis C, Kratiras Z, Samarinas M, Skriapas K (2016). Optimal bladder diary duration for patients with suprapontine neurogenic lower urinary tract dysfunction. Int Braz J Urol.

[12] Kolodynska G, Zalewski M, Rozek-Piechura K (2019). Urinary incontinence in postmenopausal women—causes, symptoms, treatment. Prz Menopauzalny.

[13] Laycock J (1994). Pelvic muscle exercises: physiotherapy for the pelvic floor. Urol Nurs.

[14] Svabik K, Hubka P, Masata J, Martan A (2018). How accurate are we in urethral mobility assessment? Comparison of subjective and objective assessment. Ceska Gynekol.

[15] Chughtai B, Hauser N, Anger J (2017). Trends in surgical management and pre-operative urodynamics in female medicare beneficiaries with mixed incontinence. Neurourol Urodyn.

[16] Verghese TS, Middleton LJ, Daniels JP, Deeks JJ, Latthe PM (2018). The impact of urodynamics on treatment and outcomes in women with an overactive bladder: a longitudinal prospective follow-up study. Int Urogynecol J.

[17] Sinha S, Lakhani D, Singh VP (2019). Cough associated detrusor overactivity in women with urinary incontinence. Neurourol Urodyn.

[18] Hu Y, Lou Y, Liao L, Xu M, Zhang H, Yang Q, Wu H. Comparison of urodynamics and perineal ultrasonography for the diagnosis of mixed urinary incontinence in women. J Ultrasound Med. 2018;37(11):2647–56. 10.1002/jum.14626 29608019

[19] Bo K, Frawley HC, Haylen BT (2017). An International Urogynecological Association (IUGA)/International Continence Society (ICS) joint report on the terminology for the conservative and nonpharmacological management of female pelvic floor dysfunction. Neurourol Urodyn.

[20] Dufour S, Wu M (2020). No. 397—conservative care of urinary incontinence in women. J Obstet Gynaecol Can.

[21] NICE (CG123) Urinary incontinence and pelvic organ prolapse in women: management. 2019. 31211537

[22] Steenstrup B, Lopes F, Cornu JN, Gilliaux M (2022). Cognitive-behavioral therapy and urge urinary incontinence in women. A systematic review. Int Urogynecol J.

[23] Imamura M, Williams K, Wells M, McGrother C (2015). Lifestyle interventions for the treatment of urinary incontinence in adults. Cochrane Database Syst Rev.

[24] Vissers D, Neels H, Vermandel A (2014). The effect of non-surgical weight loss interventions on urinary incontinence in overweight women: a systematic review and meta-analysis. Obes Rev.

[25] Todhunter-Brown A, Hazelton C, Campbell P, Elders A, Hagen S, McClurg D (2022). Conservative interventions for treating urinary incontinence in women: an overview of Cochrane systematic reviews. Cochrane Database Syst Rev.

[26] Hou Y, Feng S, Tong B, Lu S, Jin Y (2022). Effect of pelvic floor muscle training using mobile health applications for stress urinary incontinence in women: a systematic review. BMC Womens Health.

[27] Dantas LO, Carvalho C, Santos BLJ, Ferreira CHJ, Bo K, Driusso P (2021). Mobile health technologies for the management of urinary incontinence: a systematic review of online stores in Brazil. Braz J Phys Ther.

[28] Miller JM, Hawthorne KM, Park L, Tolbert M (2020). Self-perceived improvement in bladder health after viewing a novel tutorial on knack use: a randomized controlled trial pilot study. J Womens Health (Larchmt).

[29] Balk EM, Rofeberg VN, Adam GP, Kimmel HJ, Trikalinos TA, Jeppson PC (2019). Pharmacologic and nonpharmacologic treatments for urinary incontinence in women: a systematic review and network meta-analysis of clinical outcomes. Ann Intern Med.

[30] Dmochowski RR, Haab F, Robinson D (2021). A randomized, placebo-controlled clinical development program exploring the use of litoxetine for treating urinary incontinence. Neurourol Urodyn.

[31] Haab F (2019). Exploration of litoxetine (LTX): a potential novel treatment for mixed urinary incontinence (MUI). Eur Urol Suppl.

[32] Liu B, Liu Y, Qin Z (2019). Electroacupuncture versus pelvic floor muscle training plus solifenacin for women with mixed urinary incontinence: a randomized noninferiority trial. Mayo Clin Proc.

[33] Ludwig S, Becker I, Mallmann P, Jager W (2019). Comparison of solifenacin and bilateral apical fixation in the treatment of mixed and urgency urinary incontinence in women: URGE 1 study, a randomized clinical trial. In Vivo.

[34] Minagawa T, Gotoh M, Yokoyama O (2018). Therapeutic effect of propiverine hydrochloride on mixed-type urinary incontinence in women: The Female Urgency and Stress Urinary Incontinence Study of Propiverine Hydrochloride trial. Int J Urol.

[35] Najeeya AGF, Sultana A (2018). Efficacy of mace (Arils of Myristica fragrans Houtt) plus PFMT on symptoms in mixed urinary incontinence: a randomized placebo-controlled trial. Integr Med Res.

[36] Schiavi MC, D'oria O, Aleksa N (2019). Usefulness of ospemifene in the treatment of urgency in menopausal patients affected by mixed urinary incontinence underwent mid-urethral slings surgery. Gynecol Endocrinol.

[37] Shi H, Chen H, Zhang Y, Cui Y (2020). The efficacy and safety of vibegron in treating overactive bladder: a systematic review and pooled analysis of randomized controlled trials. Neurourol Urodyn.

[38] Takahashi S, Mishima Y, Kuroishi K, Ukai M (2022). Efficacy of mirabegron, a beta(3)-adrenoreceptor agonist, in Japanese women with overactive bladder and either urgency urinary incontinence or mixed urinary incontinence: post-hoc analysis of pooled data from two randomized, placebo-controlled, double-blind studies. Int J Urol.

[39] Thomas-White K, Taege S, Limeira R (2020). Vaginal estrogen therapy is associated with increased Lactobacillus in the urine of postmenopausal women with overactive bladder symptoms. Am J Obstet Gynecol.

[40] Viereck V, Gamper M, Walser C, Fesslmeier D, Munst J, Zivanovic I (2021). Combination therapy with botulinum toxin and bulking agent—an efficient, sustainable, and safe method to treat elderly women with mixed urinary incontinence. Neurourol Urodyn.

[41] Yung KK, Cheung RYK, Wan OYK, Lee LLL, Choy KW, Chan SSC (2023). Treatment outcome of women with urodynamic mixed urinary incontinence: an observational study. Int Urogynecol J.

[42] Bent AE, Gousse AE, Hendrix SL (2008). Duloxetine compared with placebo for the treatment of women with mixed urinary incontinence. Neurourol Urodyn.

[43] Schagen van Leeuwen JH, Lange RR, Jonasson AF, Chen WJ, Viktrup L (2008). Efficacy and safety of duloxetine in elderly women with stress urinary incontinence or stress-predominant mixed urinary incontinence. Maturitas.

[44] Maund E, Guski LS, Gotzsche PC (2017). Considering benefits and harms of duloxetine for treatment of stress urinary incontinence: a meta-analysis of clinical study reports. CMAJ.

[45] Porena M, Costantini E, Lazzeri M (2013). Mixed incontinence: how best to manage it?. Curr Bladder Dysfunct Rep.

[46] Kammerer-Doak D, Rizk DE, Sorinola O, Agur W, Ismail S, Bazi T (2014). Mixed urinary incontinence: International Urogynecological Association research and development committee opinion. Int Urogynecol J.

[47] Cody JD, Jacobs ML, Richardson K, Moehrer B, Hextall A (2012). Oestrogen therapy for urinary incontinence in post-menopausal women. Cochrane Database Syst Rev.

[48] Hendrix SL, Cochrane BB, Nygaard IE (2005). Effects of estrogen with and without progestin on urinary incontinence. JAMA.

[49] Grodstein F, Lifford K, Resnick NM, Curhan GC (2004). Postmenopausal hormone therapy and risk of developing urinary incontinence. Obstet Gynecol.

[50] Cipullo LM, Cosimato C, Filippelli A (2014). Pharmacological approach to overactive bladder and urge urinary incontinence in women: an overview. Eur J Obstet Gynecol Reprod Biol.

[51] Hashim H, Abrams P (2006). Pharmacological management of women with mixed urinary incontinence. Drugs.

[52] Castleden CM, Duffin HM, Gulati RS (1986). Double-blind study of imipramine and placebo for incontinence due to bladder instability. Age Ageing.

[53] Hunsballe JM, Djurhuus JC (2001). Clinical options for imipramine in the management of urinary incontinence. Urol Res.

[54] Sacomani CAR, Almeida FG, Silvinato A, Bernardo WM (1992). Overactive bladder—pharmacological treatment. Rev Assoc Med Bras.

[55] Staskin DR, Te AE (2006). Short- and long-term efficacy of solifenacin treatment in patients with symptoms of mixed urinary incontinence. BJU Int.

[56] Gomelsky A, Dmochowski RR (2011). Treatment of mixed urinary incontinence in women. Curr Opin Obstet Gynecol..

[57] Richter HE, Carnes MU, Komesu YM, Lukacz ES, Arya L, Bradley M, Rogers RG, Sung VW, Siddiqui NY, Carper B, Mazloomdoost D, Dinwiddie D, Gantz MG (2022). Association between the urogenital microbiome and surgical treatment response in women undergoing midurethral sling operation for mixed urinary incontinence. Am J Obstet Gynecol.

[58] Sung VW, Richter HE, Moalli P, Weidner AC, Nguyen JN, Smith AL, Dunivan G, Ridgeway B, Borello-France D, Newman DK, Mazloomdoost D, Carper B, Gantz MG (2021). Characteristics associated with treatment failure 1 year after midurethral sling in women with mixed urinary incontinence. Obstet Gynecol.

[59] Welk B, Baverstock RJ (2017). The management of mixed urinary incontinence in women. Can Urol Assoc J.

[60] Kershen RT, Appell RA (2002). De novo urge syndrome and detrusor instability after anti-incontinence surgery: current concepts, evaluation, and treatment. Curr Urol Rep.

[61] Zyczynski HM, Albo ME, Goldman HB, Wai CY, Sirls LT, Brubaker L, Norton P, Varner RE, Carmel M, Kim HY (2015). Change in overactive bladder symptoms after surgery for stress urinary incontinence in women. Obstet Gynecol.

[62] Ulmsten U, Henriksson L, Johnson P, Varhos G (1996). An ambulatory surgical procedure under local anesthesia for treatment of female urinary incontinence. Int Urogynecol J Pelvic Floor Dysfunct..

[63] Kenton K, Stoddard AM, Zyczynski H, Albo M, Rickey L, Norton P, Wai C, Kraus SR, Sirls LT, Kusek JW, Litman HJ, Chang RP, Richter HE (2015). 5-year longitudinal followup after retropubic and transobturator mid urethral slings. J Urol.

[64] Abdel-Fattah M, Cao G, Mostafa A (2017). Long-term outcomes for transobturator tension-free vaginal tapes in women with urodynamic mixed urinary incontinence. Neurourol Urodyn..

[65] Palomba S, Oppedisano R, Falbo A, Torella M, Maiorana A, Materazzo C, Tolino A, Mastrantonio P, La Sala GB, Alio L, Colacurci N, Zullo F (2014). Single-incision mini-slings versus retropubic tension-free vaginal tapes: a multicenter clinical trial. J Minim Invasive Gynecol.

[66] Kulseng-Hanssen S, Husby H, Schiotz HA (2008). Follow-up of TVT operations in 1,113 women with mixed urinary incontinence at 7 and 38 months. Int Urogynecol J Pelvic Floor Dysfunct.

[67] Sinha D, Blackwell A, Moran PA (2008). Outcome measures after TVT for mixed urinary incontinence. Int Urogynecol J Pelvic Floor Dysfunct.

[68] Jain P, Jirschele K, Botros SM, Latthe PM (2011). Effectiveness of midurethral slings in mixed urinary incontinence: a systematic review and meta-analysis. Int Urogynecol J..

[69] Sung VW, Borello-France D, Newman DK (2019). Effect of behavioral and pelvic floor muscle therapy combined with surgery vs surgery alone on incontinence symptoms among women with mixed urinary incontinence: The ESTEEM Randomized Clinical Trial. JAMA..

[70] Chai TC, Albo ME, Richter HE, Norton PA, Dandreo KJ, Kenton K, Lowder JL, Stoddard AM (2009). Complications in women undergoing Burch colposuspension versus autologous rectus fascial sling for stress urinary incontinence. J Urol.

[71] Lapitan MCM, Cody JD, Mashayekhi A (2017). Open retropubic colposuspension for urinary incontinence in women. Cochrane Database Syst Rev..

[72] Saraswat L, Rehman H, Omar MI, Cody JD, Aluko P, Glazener CM (2020). Traditional suburethral sling operations for urinary incontinence in women. Cochrane Database Syst Rev.

[73] Chou EC, Flisser AJ, Panagopoulos G, Blaivas JG (2003). Effective treatment for mixed urinary incontinence with a pubovaginal sling. J Urol..

[74] Itkonen Freitas AM, Mentula M, Rahkola-Soisalo P, Tulokas S, Mikkola TS (2020). Tension-free vaginal tape surgery versus polyacrylamide hydrogel injection for primary stress urinary incontinence: a randomized clinical trial. J Urol.

[75] Itkonen Freitas AM, Isaksson C, Rahkola-Soisalo P, Mentula M, Mikkola TS. Quality of life and sexual function after tension-free vaginal tape and polyacrylamide hydrogel injection for primary stress urinary incontinence: 3-year follow-up from a randomized clinical trial. Int Urogynecol J. 2023. 10.1007/s00192-023-05626-x PMC10756861 37672047

[76] Balk E, Adam GP, Kimmel H (2018). Nonsurgical treatments for urinary incontinence in women: a systematic review update.

[77] Hansen MF, Lose G, Kesmodel US, Gradel KO (2017). A national population-based cohort study of urethral injection therapy for female stress and mixed urinary incontinence: the Danish Urogynaecological Database, 2007-2011. Int Urogynecol J.

[78] Brosche T, Kuhn A, Lobodasch K, Sokol ER (2021). Seven-year efficacy and safety outcomes of Bulkamid for the treatment of stress urinary incontinence. Neurourol Urodyn.

[79] Giammo A, Geretto P, Ammirati E, Manassero A, Squintone L, Falcone M, Costantini E, Del PG, Finazzi AE, Giannantoni A, Li Marzi V, Mancini V, Musco S, Pastorello M, Pistolesi D, Risi O, Gontero P. Urethral bulking in the treatment of stress and mixed female urinary incontinence: results from a multicenter cohort and predictors of clinical outcomes. J Clin Med. 2022;11(6) 10.3390/jcm11061569 PMC8950172 35329895

[80] Hajebrahimi S, Shamsi-Sisi H, Jahantabi E, Salehi-Pourmehr H, Hashim H (2022). Efficacy of combination therapy of mid-urethral sling and low-dose abobotulinumtoxin-A injection in mixed urinary incontinence. Neurourol Urodyn..

[81] Komar A, Bretschneider CE, Mueller MG, Lewicky-Gaupp C, Collins S, Geynisman-Tan J, Tavathia M, Kenton K. Concurrent retropubic midurethral sling and onabotulinumtoxina for mixed urinary incontinence: a randomized controlled trial. Obstet Gynecol. 2021;137(1):12-20. 10.1097/AOG.0000000000004198 33278293

[82] Chang YH, Hsiao PJ, Chi-Ping H, Wu HC, Hsieh PF, Chou EC. A comparative observational study to evaluate the efficacy of mid-urethral sling with botulinum toxin a injection in urinary incontinence patients. Toxins (Basel). 2020;12(6) 10.3390/toxins12060365 PMC7354459 32498306

[83] Ogrinc UB, Sencar S, Lenasi H (2015). Novel minimally invasive laser treatment of urinary incontinence in women. Lasers Surg Med.

[84] Okui N, Miyazaki H, Takahashi W, Miyauchi T, Ito C, Okui M, Shigemori K, Miyazaki Y, Vizintin Z, Lukac M (2022). Comparison of urethral sling surgery and non-ablative vaginal Erbium:YAG laser treatment in 327 patients with stress urinary incontinence: a case-matching analysis. Lasers Med Sci.

[85] Kang J, Sun Y, Su T, Liu Y, Liang F, Liu Z (2021). Electroacupuncture for balanced mixed urinary incontinence: secondary analysis of a randomized non-inferiority controlled trial. Int Urogynecol J..

[86] Sun Y, Liu Y, Chen H, Yan Y, Liu Z (2021). Electroacupuncture for stress-predominant mixed urinary incontinence: a protocol for a three-armed randomised controlled trial. BMJ Open..

[87] Ayeleke RO, Hay-Smith EJ, Omar MI (2015). Pelvic floor muscle training added to another active treatment versus the same active treatment alone for urinary incontinence in women. Cochrane Database Syst Rev..

[88] Carletti V, Yacoub V, Grilli D, et al. Sequential combined approach in patients with mixed urinary incontinence: surgery followed by posterior tibial nerve stimulation. Minerva Obstet Gynecol. 2022. 10.23736/S2724-606X.22.05106-5. 10.23736/S2724-606X.22.05106-5 35785925

[89] Long Z, Chen H, Yu S, Wang X, Liu Z (2022). Effect of acupuncture for mixed urinary incontinence in women: a systematic review. Front Public Health..

